# No evidence that *ACE2* or *TMPRSS2* drive population disparity in COVID risks

**DOI:** 10.1186/s12916-024-03539-0

**Published:** 2024-08-26

**Authors:** Nathaniel M. Pearson, John Novembre

**Affiliations:** 1 Root Deep Insight, Boston, MA USA; 2 Department of Human Genetics, University of Chicago, Chicago, IL USA

**Keywords:** *ACE2*, COVID, COVID19, Functional prediction, GWAS, Host genetics, Human genes, Immunity, Infection, Polygenic risk, Population genetics, Population structure, Rare variants, Sample design, Sample size, Sampling, SARS-CoV2, *TMPRSS2*

## Abstract

Early in the SARS-CoV2 pandemic, in this journal, Hou et al. (BMC Med 18:216, 2020) interpreted public genotype data, run through functional prediction tools, as suggesting that members of particular human populations carry potentially COVID-risk-increasing variants in genes *ACE2* and *TMPRSS2* far more often than do members of other populations. Beyond resting on predictions rather than clinical outcomes, and focusing on variants too rare to typify population members even jointly, their claim mistook a well known artifact (that large samples reveal more of a population’s variants than do small samples) as if showing real and congruent population differences for the two genes, rather than lopsided population sampling in their shared source data. We explain that artifact, and contrast it with empirical findings, now ample, that other loci shape personal COVID risks far more significantly than do *ACE2* and *TMPRSS2*—and that variation in *ACE2* and *TMPRSS2* per se unlikely exacerbates any net population disparity in the effects of such more risk-informative loci.

## Background

In mid-2020, concurrent with early empirical inquiry into roles of host genomic variation in SARS-CoV2 infection [1–5], Hou et al. set out to offer predictive guidance for such efforts, by assessing pre-pandemic public DNA data from two human genes, *ACE2* and *TMPRSS2*, whose protein products were known to interact with other coronaviruses [6].

Pooling public genotype data sampled from various human groups, without phenotypes, they shortlisted *ACE2* and *TMPRSS2* variants that some computational heuristics predicted likely to alter protein function, and found that most such variants (each, typically, very rare) came from subsets of data labeled “African/African-American” or “Non-Finnish European” versus labeled otherwise (e.g., “East Asian”).^1^ This, they held, suggested real-world population disparities in *ACE2* and *TMPRSS2* functional variant load, similar for both genes, that might in turn drive population differences in COVID outcomes.

## Errant interpretation of genotype data

Alas, Hou et al. had neglected a basic feature of the public data they used—lopsided population sample sizes—that made their summary findings artifactually likely even with no difference between real populations. Specifically, they had pooled genotypes from > 36,000 “non-Finnish European” and > 23,000 “African/African-American” people, but far fewer “Amish” (450), “Ashkenazi” (1662), “East Asian” (1567), or other (< 15,000) people.^2^ As such, even if variants were uniformly distributed across real populations, Hou et al. would likelier find a given rare variant in either of their big samples (“African/African-American” or “non-Finnish European”) than in any of their much smaller samples of other groups.

**Fig. 1:**
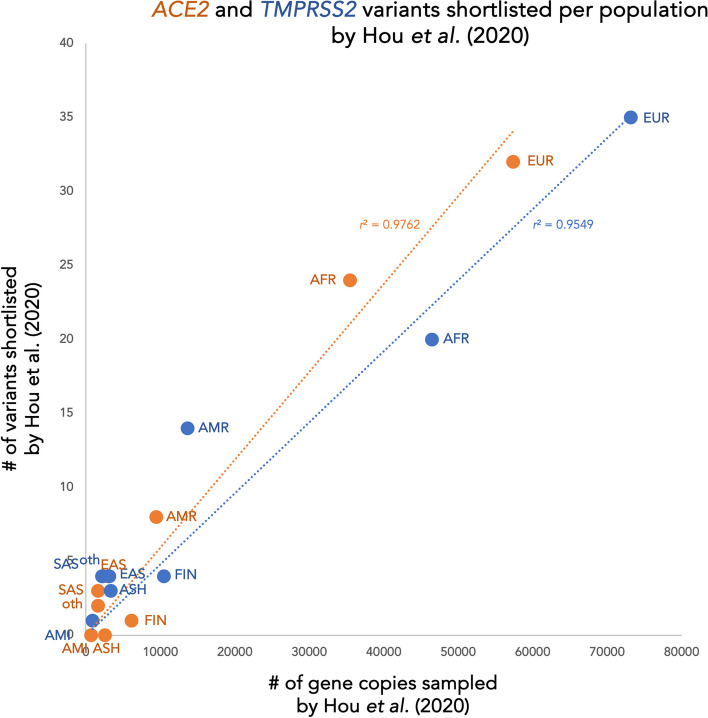
Population-specific variant tallies in Hou et al. [6] reflect lopsided sampling. Scatterplots of population-specific tallies (y-axis) of shortlisted variants in *ACE2* (orange) or *TMPRSS2* (blue), by sample size (x-axis; values denote maximum sampled alleles among shortlisted variant-position genotypes for that gene in gnomAD (v.3.0) + NHLBI-GO ESP6500 genotypes, as pooled by Hou et al. [6]). Datapoints mark values for African/African-American (AFR; *TMPRSS2* count excludes 1 variant (p.Pro444Leu) reported by Hou et al., but not in public data, and not consistent with reference variant at given protein residue); Amish (AMI); Ashkenazi (ASH); east Asian (EAS); south Asian (SAS); non-Finnish, non-Amish, non-Ashkenazi European (EUR; *TMPRSS2* count excludes 1 variant (p.Gly6Arg) reported by Hou et al., but not in public data, and not consistent with reference variant at given protein residue); Finnish (FIN in Hou et al. [6]); Latino/Admixed American (AMR; *ACE2* count includes 2 variants wrongly omitted from this population by Hou et al.); or other (oth; PNA in Hou et al. [6]; *ACE2* count excludes 2 variants wrongly tallied in this population by Hou et al.). Best-fit trends (dashed) mark origin-rooted linear regression, conservatively proxying independent (versus cumulative) discovery of potentially selection-constrained (versus selectively neutral) variants in samples from variably sized, mutually diverged populations (versus one steady-sized randomly mating population). We note that even in the contrasting case of cumulative discovery in a steady-sized population, variants under selective constraint (as Hou et al. sought to tally) tend to accrue quasi-linearly, rather than strictly logarithmically, with increasing overall sample size [8–11]

Consistent with such artifact, the number of *ACE2* or *TMPRSS2* variants Hou et al. shortlisted for a given population scales well with how many genotypes they sampled from that population (Fig. 1, origin-rooted linear *r*^2^ > 0.95 for both genes).^3^^,^^4^ Long known in theoretical and empirical population genetics [7, 11], the sampling effect apparent in Hou et al.’s summary findings reflects a simple fact: much as counting more of a forest’s birds can help document rare taxa living there, sequencing more of a population’s gene copies helps document rare variants among them. While other factors shape the emergence and fate of such variants, and their rate of discovery with increasing sample size [7–10, 12, 13], reliably finding and quantifying them entails sampling from many individuals.

As such, current public genotype data inherently document more of the variants actually circulating in some populations than of those actually circulating in other populations—and allow more precise estimation, in the same best-sampled (if at all) groups, of each variant’s actual frequency (as may figure in functional prediction) or absence. For example, to be 90% confident that even the least rare shortlisted *ACE2* variant in non-Finnish, non-Amish, non-Ashkenazi European sample data (p.R219C) is not actually more common in Amish, Ashkenazi, and/or Finnish populations, despite its absence in those groups’ small sample data, would require sampling > 22,700 additional Amish, Ashkenazi, and Finnish copies of *ACE2*.^5^

**Fig. 2:**
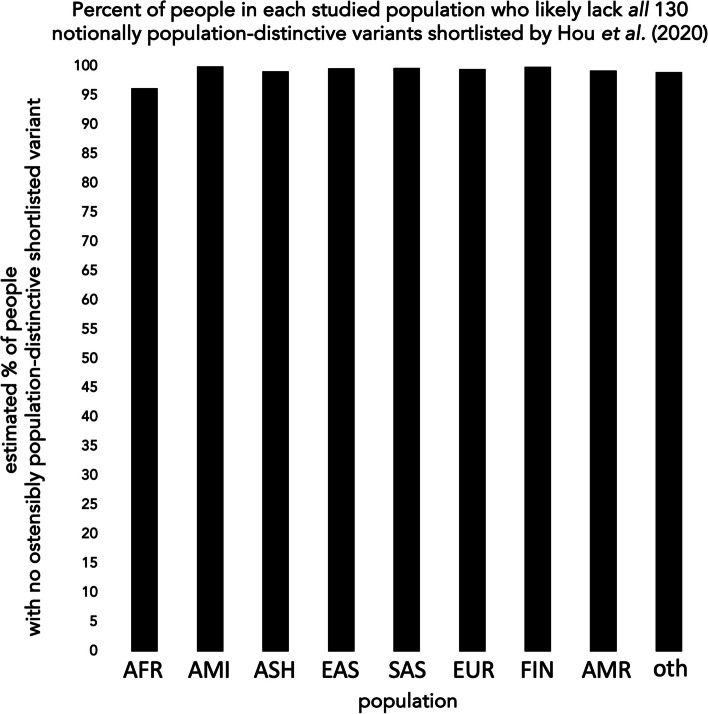
Nearly everyone, in all studied populations, likely lacks all ostensibly population-distinctive variants shortlisted by Hou et al. [6]. Bar plot of estimated percentage of people in each studied population who likely have none of the 130 notionally population-distinctive (i.e., absent in sample data from at least one studied population) *ACE2* and *TMPRSS2* variants shortlisted (without empirical evidence for any effect on protein function or other physiology, and omitting many other potentially functionally relevant variants in all populations) by Hou et al. [6]. Estimates (product of binomial probabilities) presume variants assort randomly, independently, at sampled population-specific frequencies, in half-XX/half-XY populaces. AFR = African/African-American; AMI = Amish; ASH = Ashkenazi; EAS = east Asian; SAS = south Asian; EUR = non-Finnish, non-Amish, non-Ashkenazi European; FIN = Finnish; AMR = Latino/Admixed American; oth = other. Values may underestimate true minimum region-wide percentage, as (i) the least rare such variant (*ACE2* p.L731F), which most strongly suppresses the AFR estimate, appears mainly in data from over-proportionately sampled west Africa, more so than in data yet sampled from likewise populous peoples elsewhere in Africa and diaspora [17]; and (ii) any pairwise linkage among shortlisted variants would increase the proportion of people inheriting neither variant in such pairs

Beyond summary tallies, none of Hou et al.’s shortlisted variants reliably proxies any population to begin with. One (*TMPRSS2* p.V160M) appears in all studied populations—and in many individuals in each—so offers scant ground to guess which population(s) a carrier comes from. All 130 other shortlisted variants appear too rare in every population to typify members of any of them (even in aggregate, their data suggest that > 96% of people in every studied population likely carry none of those rare variants (Fig. 2)).^6^ And as real populations also harbor unsampled but functionally relevant variants, whose effects on basic protein function (let alone response to a particular virus) current heuristics cannot reliably predict [14–16], the tallies and predictions of Hou et al. do not warrant positing that ACE2 or TMPRSS2 (let alone both) functions worse with respect to SARS-CoV2 in any human population (let alone particular ones) than in others.

In principle, real populations may indeed differ, if modestly, in functionally relevant patterns of variation in a gene (or even genome-wide), pending their histories. All else equal, for example, big populations tend to accrue and keep more genetic variation (especially if non-harmful) than do small populations [12, 18, 19]; fast-growing populations to accrue more new rare variation in particular [9, 20]; and genome segments under strong directional selection, in a given population’s environment, may (along with flanking segments coupled to them without recombination) tend to lose such rare variation in particular [10].

But as human genes thus vary in summary patterns of variation, via potentially population-distinctive histories, meaningfully comparing such patterns requires sampling well, and assessing not just which variants appear at all in data sampled from a population, but the summary distributions of their estimated frequencies (respective proportions of sampled gene copies harboring them).

To that end, frequency-sensitive summary metrics^7^ show less variation in human *ACE2*, both within and between most human populations,^8^ than for most other X-borne^9^ or autosomal [21, 22, 28, 29] human genes, limiting the extent to which populations’ distinctive histories may yield disparate patterns of variation. By comparison, such well grounded summary metrics show more overall variation in human *TMPRSS2* [21]—much of it shared across populations, in varied patterns that reflect the cross-regional spread of variants old (and generally non-harmful) enough to have become common.

Importantly, even beyond the two genes’ contrasting patterns of variation, pandemic-long cohort outcomes have not shown variation in either *ACE2* or *TMPRSS2* to shape personal COVID risks nearly as significantly as variation elsewhere in our genomes—including the most strongly and significantly risk-shaping locus, on the short arm of chromosome 3; the *ABO* blood group locus on chromosome 9; and other autosomal loci [1–5, 17, 30]. Some non-protein-altering variants in *ACE2* and *TMPRSS2* have met multiple-test-stringent significance criteria for association with risks of SARS-CoV2 infection (an *ACE2* regulatory variant cluster) or severity (*TMPRSS2* intronic variant), but their significance falls short of that evident for other loci. And among variants shortlisted by Hou et al., only one (the relatively common *TMPRSS2* p.V160M) has shown even suggestive (not multiple-test-stringent) evidence for association with any COVID risk [30–33]—while broader tests, tuned and powered specifically to detect rare variant association per se in clinically characterized population cohorts, have not implicated shortlisted or other rare protein-coding variation in either gene in COVID risks [34, 35].

Moreover, across all COVID-implicated loci, risk-informative variants differ in their population distributions and inferred effects, in many cases in partial counter-balance to one another. The *ACE2*-regulatory rs190509934C variant, for example, associates significantly with below-average risk of SARS-CoV2 infection (and suggestively with below-average risk of severe COVID), but appears least rare in a studied broad population (south Asian) in which other loci harbor variant loads most strongly associated with above-average risk of infection (and severe disease) [36, 37].

Altogether, such mixes of above- and below-average risk-associated variants in human genomes worldwide leave a broad range of risk-relevant personal variant load within every studied human population [38–41]. And those loads, in turn, explain < 10% of personal variability in COVID risks evident, so far, in clinically characterized cohorts [39], while other factors, such as age, background health, and immune exposures, show far stronger effects.^10^ As such, now-ample data suggest that COVID incidence and severity likely differ among human populations far less by genetics than by factors such as age structure, past and ongoing immune exposures, comorbidity prevalences, and access to effective health interventions [4, 17, 30, 39, 40, 45–52]—and pointedly, do not support speculation that variation in *ACE2* or *TMPRSS2* drives net population disparity in genetically attributable (or overall) COVID risks.^11^

## Conclusions

Speculating early in the pandemic, on potential COVID-relevance of variation in two of humanity’s many genes, Hou et al. understandably settled for predictive heuristics in lieu of clinical data. But in tallying shortlisted *ACE2* and *TMPRSS2* variants among populations, they mistook a sample size artifact as if evidence of population differences—and tried to proxy population-representative gene function by tallying shortlisted variants found at all, instead of summing empirically estimated, genotype frequency-scaled effects. Though their shortlist offered well intended, if unvalidated, candidates for early-pandemic study, genome-wide empirical insights have eclipsed its utility—while leaving, unaddressed, their artifactual summary claims. And those claims, in turn, have drawn credulous citation in public discourse, hindering understanding of COVID risks and of human genetic diversity itself [57–59].

While pitfalls of methodology and interpretation have long plagued basic and clinical research [60–67], public discourse invoking Hou et al. [6] highlights how platforms to usefully share and discuss such research can also virally spread faulty inferences missed by authors and reviewers, misleading not just researchers and clinicians, but also lay-people who may rely on published science in personal, professional, family, and civic decisions [64]. As such, we hope that correcting basic errors that misled Hou et al., and those citing their work, helps right the record on human genomic variation in *ACE2*, *TMPRSS2*, and loci more informative of COVID risks—and, further, encourages critical stringency in the interpretation of population genetic and other data, amid efforts [68, 69] to more promptly and soundly validate research published, and subsequently invoked, in and between societal crises.

## Data Availability

Datasets analyzed in this study are available at: • https://cloud.google.com/life-sciences/docs/resources/public-datasets/gnomad (gnomAD v3.0 data includes 1000Genomes data, as analyzed by Hou et al.) • https://esp.gs.washington.edu/drupal/dbGaP_Releases • https://www.covid19hg.org/

## Abbreviations

*ACE2*: Angiotensin converting enzyme 2 [gene]
AMI: Amish
AMR: [Latina/o] Admixed American
ASH: Ashkenazi
COVID: Coronavirus disease
DNA: Deoxyribonucleic acid
EAS: East Asian
EUR: [Non-Amish, non-Ashkenazi, non-Finnish] European
FIN: Finnish
oth: Other [population(s)]
SARS-CoV2: Severe acute respiratory syndrome coronavirus 2
SAS: South Asian
*TMPRSS2*: Transmembrane serine protease 2 [gene]

## Footnotes

1 Denoted by Hou et al. as, respectively, “African/African-American” and “Non-Finnish [and implicitly non-Amish, non-Ashkenazi] European” in Hou et al., these classifications group genetically and culturally disparate peoples, across wide modern and ancestral geographic ranges, at different scales, under single umbrella terms.

2 Compounding their lopsided sampling, Hou et al. also reported adding, to the foregoing totals, duplicate copies of many non-Amish, non-Ashkenazi genotypes in particular (from the 1000 Genomes Project dataset, which their main gnomAD v3.0 data already included), further confounding any frequency estimation of individual variants.

3 While cumulative discovery of selectively neutral variants, in a steady-sized randomly mating population, tends to grow logarithmically with sample size [7], Hou et al. instead tallied selection-liable (protein-altering) variants, non-cumulatively (i.e., via independent single samplings) from mutually diverged, variably sized, and potentially variably growing populations. The foregoing factors more conservatively suit linear than logarithmic (or other presumptive higher-order) regression [8–10].

4 Further, each *ACE2* variant tally, and all but two *TMPRSS2* variant tallies (those for the highly recently admixed “Admixed Latino” and “Population Not Assigned” samples), accords the 95%-confident expectation for a random sample of its respective size, without replacement, from total genotypes studied for that gene, given total shortlisted variant incidences.

5 Confidence bound presumes accurate estimate of variant frequency in non-Finnish, non-Amish, non-Ashkenazi Europeans, and large randomly mating populations such that sampling probabilities approximate binomial expectation.

6 Estimates (products of binomial probabilities) presume that variants assort randomly, independently, at sampled population-specific frequencies, in half-XX/half-XY populaces.

7 For example, copy-pairwise per-site mismatch probability (nucleotide diversity), Tajima’s *D*, and Wright’s *F*_*ST*_.

8 Save for in mutually neighboring Baka, Bakola, and Bedzan peoples, among whom three protein-coding variants rare or absent in other central African and broader populations have become moderately common (perhaps via shared linkage to regulatory variants favored by local selection over many past generations) [21]. A smaller survey [22] that reported some metrics in European-Americans suggested excess intermediate-frequency variation; but those metrics tend to spuriously infer such skew in small samples [23]. By contrast, in the same study [21], metrics robust to sample size [23] affirmed less overall variation in *ACE2* than in other X-linked genes.

9 Overall, X-specific genes such as *ACE2* tend, theoretically and empirically, to vary less among people overall or within a particular population, but more (relative to such scant overall variation) between randomly chosen members of different populations, than do autosomal genes [24–27].

10 Even for severe COVID (Hou et al.’s focus), sensitive liability estimates [42] at conservatively estimated population prevalences [43] were < 10% [30]. One non-genetic (pedigree-based) survey suggested possibly greater heritability, still surpassed by inferred effects of residence environment alone, but in lacking genetic data, could not control for close-kin or/and family size biases in exposures within, between, and beyond residences (e.g., in room-sharing, social/care visitation, and/or similarity of work), or for family-wise differences in age or non-residence environmental exposures [44]. And on the specific question of population (versus personal or family) disparity in inborn severe COVID risk, early speculation from sparsely typed small samples [38] has not borne out in well powered cohort data [39, 45].

11 Specifically, empirically estimated variance in population-mean genomically attributable risk (the summed product of significantly risk-associated variants’ sampling-inferred genotype frequencies and empirically estimated additive risk contributions [36]), for the focal outcome of Hou et al. (severe COVID) [6], in the same populations they studied, is slightly greater (~ 0.02089) when omitting *ACE2* variation than when including it (~ 0.02004) or when also including *TMPRSS2* variation (0.01822) [37]. These estimates warrant much caution, as variant-attributable effects themselves vary among populations [30] (plausibly via population-stratified genome-wide variation, local haplotype structure, environmental modifiers [53–56], and other factors), but suggest that, all else equal, *ACE2* and (if relevant) *TMPRSS2* variation unlikely exacerbates any net disparity in such additively genomically attributable COVID risk among studied human populations.
